# Preferences for befriending schemes: a survey of patients with severe mental illness

**DOI:** 10.1186/s12888-018-1643-9

**Published:** 2018-03-09

**Authors:** Sarah Toner, Megan Cassidy, Agnes Chevalier, Aida Farreny, Monica Leverton, Mariana Pinto da Costa, Stefan Priebe

**Affiliations:** 1 Unit for Social and Community Psychiatry (WHO Collaborating Centre for Mental Health Service Development), Newham Centre for Mental Health, Queen Mary University of London, London, UK; 2 Hospital de Magalhães Lemos, Porto, Portugal; 3 Institute of Biomedical Sciences Abel Salazar (ICBAS), University of Porto, Porto, Portugal

**Keywords:** Befriending, Preference, Mental illness, Loneliness, Social isolation, Social contacts, Quality of life

## Abstract

**Background:**

Befriending has become a widely used method for tackling social isolation in individuals with severe mental illness (SMI), and evidence exists to support its effectiveness. However, patient preferences for befriending remain unclear. We aimed to determine whether patients with SMI want a volunteer befriender and, if so, the volunteer characteristics and character of the relationship they would prefer.

**Methods:**

A survey of outpatients was conducted across London-based community mental health teams, for individuals diagnosed with affective or psychotic disorders. Questions consisted of measures of demographic characteristics, befriending preferences and social context, including measures of time spent in activities, number of social contacts, loneliness and subjective quality of life (SQOL). Binary logistic regressions were used to investigate potential predictors of willingness to participate in befriending.

**Results:**

The sample comprised of 201 participants with a mean age of 43 years. The majority (58%) of the sample indicated willingness to participate in befriending. In univariable analyses this was associated with less time spent in activities in the previous week, higher level of loneliness and lower SQOL. When all three variables were tested as predictors in a multivariable analysis, only lower SQOL remained significantly associated with willingness to take part in befriending.

Relative to other options presented, large proportions of participants indicated preference for weekly (44%), 1-hour (39%) meetings with a befriender, with no limits on the relationship duration (53%). Otherwise, patient preferences exhibited great variability in relation to other characteristics of befriending schemes.

**Conclusions:**

A substantial number of patients with SMI appear willing to take part in a befriending scheme. Patients with lower SQOL are more likely to accept befriending, so that befriending schemes may be a realistic option to help patients with particularly low SQOL. The large variability in preferences for different types of befriending suggests that there is no one-size-fits-all formula and that schemes may have to be flexible and accommodate different individual preferences.

## Background

Individuals with severe mental illness (SMI) reportedly experience higher levels of social isolation and loneliness than the general population, which is linked with low levels of social support [1, 2]. Isolation and loneliness negatively impact on both physical and mental health [3], and potentially exacerbate pre-existing mental health problems. Such patterns may be seen in patients with schizophrenia; negative symptoms may include social withdrawal and blunted emotional responses, which persist even in stable patients [4]. Such symptoms may both increase the likelihood and worsen the impact of social isolation.

Social isolation experienced by individuals with SMI can be tackled through interventions from volunteering organisations, representing an important resource for health services when utilised efficiently [5]. Volunteers often provide support and help to build closer relationships with both services and communities [6]. One example of this is a type of volunteering termed ‘befriending’. This denotes a one-to-one companionship facilitated by a volunteering organisation or a health or social service to foster a supportive relationship between a volunteer and an individual with a physical or mental disorder.

Befriending remains poorly understood and under researched with regard to the extent and nature of its impact on patient outcomes [7, 8]. Systematic reviews show a shortage of high quality studies [2, 9]. Based on existing studies, the reviews report a tangible benefit of befriending on depressive symptoms [2] and for both physical and mental health disorders [9]. Siette and colleagues [9] highlighted existing limitations within befriending practices, including the need to better define appropriate populations for befriending schemes. The authors assert that greater efficacy may be achieved through balancing the frequency, length and modality of befriending. This process may be enriched by input from patients, which is currently lacking. This is particularly relevant given that patients with SMI experiencing negative symptoms may be particularly hard to reach through befriending schemes.

Although befriending can be used to support patients with SMI, little is known about their preferences with regard to the aim of befriending, structure of the scheme and its activities, or the nature of the befriender-patient relationship. This conflicts with the growing focus on patient perspectives in informing their care, which has been sought-after and encouraged in health services [10, 11]. Identifying and addressing the preferences of those patients who are particularly challenging to recruit into social interventions, such as those with pronounced negative symptoms, particularly relevant if these schemes are to be successful in targeting social isolation.

Through a survey of current patients with SMI, we firstly aimed to determine if patients would like to take part in a befriending scheme. We secondly wanted to ascertain whether their willingness to become a befriendee can be predicted by patient characteristics or measures of social context relating to isolation and social activity. Thirdly, we aimed to explore what type of befriending scheme patients would prefer, if one was offered. In doing so, we intended to provide guidance for the development of future befriending schemes and help to improve the effectiveness of existing schemes.

## Method

This study aimed to determine whether patients with SMI want to participate in a befriending scheme and, if so, what volunteer and relationship characteristics they prefer.

This was a cross-sectional survey study conducted in patients from nine community mental health teams across East London. We collected data from patients about their sociodemographic characteristics, befriending preferences and social context. A favourable opinion was given by the NHS Research Ethics Committee (Ref 14/SW/1011).

### Eligibility criteria

To be deemed eligible, patients were required to meet the following criteria: (1) Aged over 18 years; (2) under the care of secondary mental health services; (3) diagnosed with a psychotic (F20-F29) or an affective disorder (F30-F39) according to the International Statistical Classification of Diseases and Related Health Problems - Tenth Revision (ICD-10), 5th edition [12]; (4) sufficient proficiency in spoken English to understand the consent process and answer the questionnaire items. Patients were excluded if deemed by their clinician to have insufficient capacity to consent at the time of assessment, or if the patient was participating in a befriending research trial, which was ongoing at the time of the assessments.

### Procedure

The research team identified eligible patients in nine community mental health teams in East London. Eligible patients were then approached on the day of their appointment with a clinician to explain the study’s aims, provide more information if requested and obtain informed written consent prior to assessment. All interviewers were trained researchers.

### Instruments

The questionnaire obtained data on patients’ socio-demographic characteristics including age, gender, years since first diagnosis of mental illness, weekly hours in employment, and monthly income of patients. This was followed by a brief description of befriending and questions relating to preferences for such schemes. Participants were first asked whether they had heard of befriending prior to the questionnaire and if they would take part in befriending if a scheme were advertised. The participants who expressed willingness were then asked a series of multiple-choice questions regarding their preferences for the characteristics of a volunteer befriender, as well as the type of befriending relationship (e.g. meeting frequency, meeting and relationship duration, and potential activities). In an open-ended question, patients who indicated that they would not like to take part in befriending were asked to give a reason for their answer.

Measures of social context assessed time spent in leisure activities (time use), social contacts, loneliness and quality of life. Time use was measured using an adapted version of the United Kingdom Time Use Survey [13], which listed potential activities and asked patients how many times the interviewee had engaged in the activity in the week prior to assessment, how long was spent doing the activity and whether this was with another person or alone [14]. Social contacts were measured using the Social Contacts Assessment [15] which asks participants to list anyone with whom they have had a social contact in the previous week, excluding people they live with or health professionals. Loneliness was rated on an item from the World Health Organization Quality of Life Assessment [16] asking “How lonely do you feel in your life?” with response options ranging from 1 (not at all) to 5 (extremely). Finally, subjective quality of life (SQOL) was assessed using the Manchester Short Assessment of Quality of Life (MANSA) [17], which asks participants to rate satisfaction with 12 life domains on a scale from 1 (couldn’t be worse) to 7 (couldn’t be better). The mean score of those 12 items is used to reflect SQOL.

### Sample size and data entry

The sample size in this exploratory study was based on an estimate for a between-group comparison of two groups. Comparing two groups with a sample size of 100 provides 80% power to detect an effect size of 0.30 at a 5% significance level. Therefore, we aimed to recruit a total sample of 200 patients.

Data were entered into SPSS (version 24) for analysis [18]. Further, 20% of the data were randomly selected, stratified according to the researcher who originally entered the data, re-entered and compared against the database to increase reliability of the data entry procedure.

### Analysis

Characteristics of the whole sample and the befriending preferences of those patients who expressed a willingness to take part in befriending were represented by descriptive outputs. Open questions were analysed using content analysis.

A univariable binary logistic regression was used to explore whether the following patient characteristics were related to the dependent variable, i.e. willingness to engage in a befriending scheme: age, gender, familiarity with befriending schemes, diagnostic group (F2 or F3), years since diagnosis, weekly hours spent in employment, monthly income, weekly time use, social contacts in the past week, loneliness and SQOL. Variables with a significant association with the willingness to take part in befriending (*p* < 0.1) were then entered in a multivariable binary logistic regression model. Patients with missing data for any variables presented in the results were excluded from the relevant analysis.

## Results

**Fig. 1:**
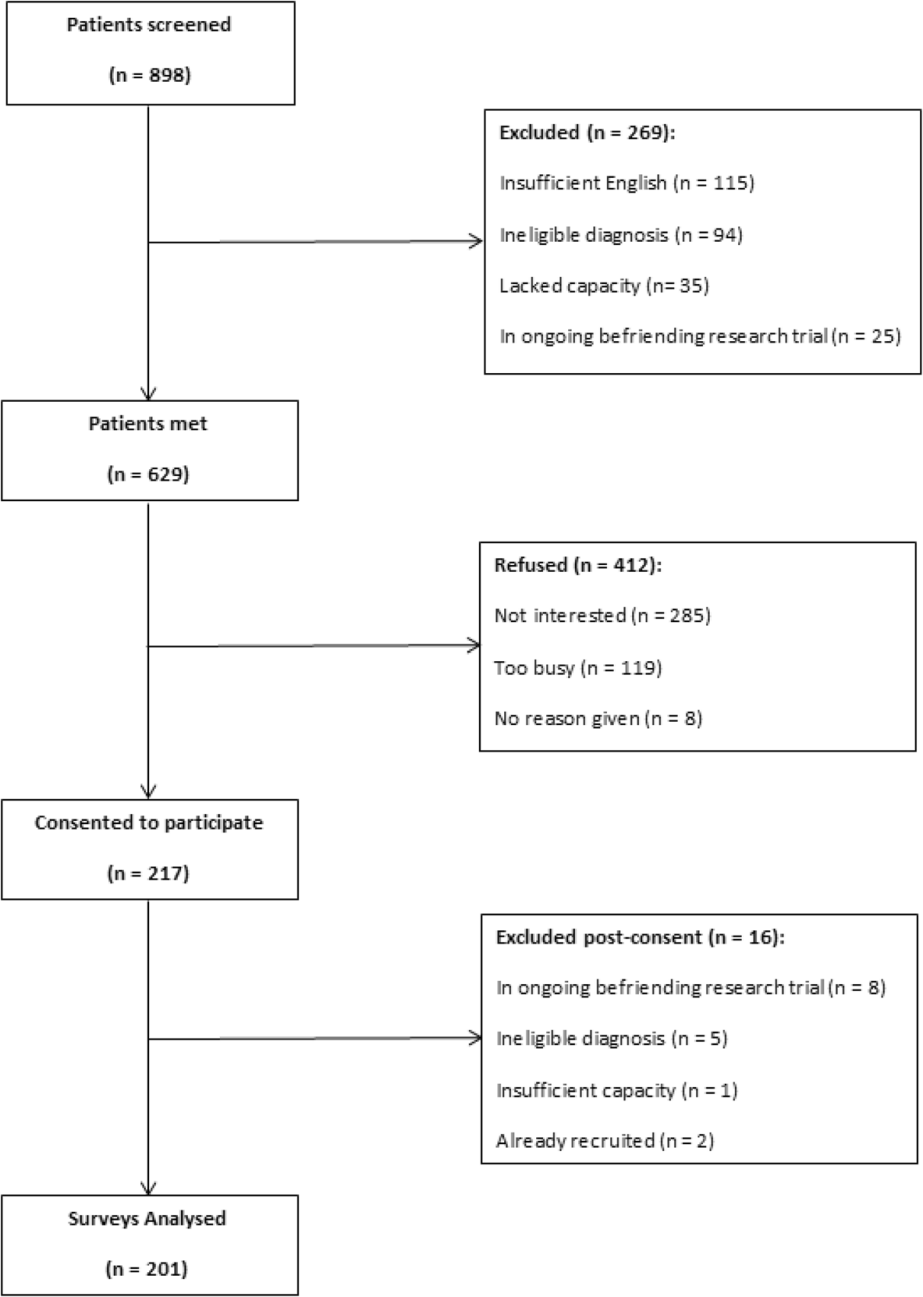
CONSORT diagram of patients approached to participate

Of the 898 patients screened, 201 were included in the final analysis. The selection process is detailed in Fig. 1.

### Sample characteristics

Sample characteristics are shown in Table 1. Approximately three quarters of the sample had been diagnosed with schizophrenia or related disorder (75%, *N* = 151) and the remainder were diagnosed with an affective disorder.

**Table 1:** Demographic characteristics, SQOL, loneliness and time use of sample

Characteristic	Sample (*N* = 201)
Age, M (SD)	43.4 (10.7)
Gender (Male), N (%)	132 (65.7)
Years since diagnosis, M (SD)	15.3 (9.7)
Ethnicity, N (%)	
White	55 (27.4)
Black African	36 (17.9)
Black Caribbean	28 (13.9)
Bangladeshi	20 (10.0)
Black Other	15 (7.5)
Pakistani	10 (5.0)
Indian	6 (3.0)
Chinese	2 (1.0)
Other	29 (14.4)
Employment, N (%)	
Unemployed	167 (83.5)
Paid employment	16 (8.0)
Training/education	5 (2.5)
Retired	6 (3.0)
Other	6 (3.0)
Hours spent in employment (weekly), M (SD)	2.2 (7.7)
Monthly Income (£), M (SD)	776.9 (516.7)
Time Use (Hours)^a^, M (SD)	3.1 (3.7)
Social Contacts^b^, M (SD)	2.0 (2.2)
Loneliness^c^, M (SD)	2.6 (1.4)
Subjective Quality of Life (MANSA)^d^, M (SD)	4.4 (1.1)

^a^Time spent doing activities over the week prior to interview

^b^Number of social contacts in week prior to assessment, either face-to-face or via technology

^c^Scored from 1 (Not at all) to 5 (Extremely)

^d^Satisfaction ratings range from 1 (couldn’t be worse) to 7 (couldn’t be better)

### Willingness to participate in a befriending scheme

Overall 116 (58%) participants indicated a willingness to take part in a befriending scheme. Of these, 51 had never heard of befriending prior to the assessment, from a total of 85 who had no prior knowledge of befriending (42% of the sample).

Of the 84 unwilling individuals, reasons for not wanting to take part fell into the following categories: befriending is not relevant to their social needs (47%, *N* = 34); lack of interest or time (26%, *N* = 19); aversion to the label of mental illness (19%, *N* = 14); and general interest but only under different personal circumstances (e.g. if mental health deteriorated) (8%, *N* = 6).

#### Predicting willingness

The univariable regression models for willingness to participate in befriending are shown in Table 2. Loneliness, number of social contacts and SQOL were significantly associated with willingness to take part in befriending. In the multivariable regression only SQOL remained significant at the 5% level, with lower SQOL indicating greater inclination to take part in a befriending scheme.

**Table 2:** Univariable logistic regression model for willingness to engage in befriending

Variables	Univariable analysis			Multivariable analysis		
	OR	(95% CI)	*P*-value	OR	(95% CI)	*P*-value
Age	1.000	(0.974 to 1.026)	.983	–	–	–
Gender (male)	0.835	(0.461 to 1.512)	.551	–	–	–
Heard of befriending (Y)	1.213	(0.685 to 2.147)	.508			
Diagnostic Group (F3)	1.000	(0.523 to 1.913)	1.00			
Years since diagnosis	1.003	(0.974 to 1.033)	.852	–	–	–
Weekly working hours	0.977	(0.942 to 1.014)	.229	–	–	–
Monthly Income	1.000	(0.999 to 1.001)	.512	–	–	–
Time use (hours)	0.998	(0.924 to 1.077)	.951	–	–	–
Social Contacts	0.876	(0.770 to 0.998)	.046	0.884	(0.774 to 1.011)	.072
Loneliness	1.387	(1.112 to 1.729)	.004	1.220	(0.945 to 1.577)	.128
SQOL	0.627	(0.469 to 0.838)	.002	0.707	(0.506 to 0.990)	.043

### Befriending preferences

Almost two-thirds of the 116 participants indicating willingness to take part in befriending stated that their main aim would be to make a new friend (62%, *N* = 71) rather than the alternative option presented (to do more activities).

#### Characteristics of volunteer befriender

Approximately one-third of patients had no preference (34%, *N* = 39) while 29% (*N* = 33) wanted someone similar to them and 29% (*N* = 33) would prefer a psychology or counselling student. Only 9% (*N* = 10) of respondents would prefer being matched with someone different to them.

Further, 70% (*N* = 81) of patients would want the volunteer befriender to be in contact with their mental health care team. A majority (62%, *N* = 72) also reported that they would prefer a befriender with lived experience as a patient in mental health care, while 21% (*N* = 24) of individuals had no preference, and 17% (*N* = 20) explicitly said they would not want a befriender with lived experience.

#### Type of the relationship with the befriender

The preferred relationship with a volunteer befriender was most frequently to have ‘a real friendship’ (32%, *N* = 37), followed by ‘someone who talks with me and listens’ (30%, *N* = 35), ‘someone who does activities with me’ (20%, *N* = 23), ‘someone to spend time with’ (11%, *N* = 13) and ‘no preference’ (7%, *N* = 8).

When offered a list of potential activities to do with a befriender more than half of participants asked selected the following, regardless of their primary aim for taking part: eating in a café or restaurant (70%, *N* = 81), just getting out of the house (61%, *N* = 70), going to the cinema or theatre (57%, *N* = 66), gym/playing sports (64%, *N* = 73), going for walks (72%, *N* = 83) and shopping (58%, *N* = 67). Further, 42% (*N* = 47) felt the scheme should cover the costs of these activities, while 32% (*N* = 35) stated the scheme should make a partial contribution, and the remaining 26% (*N* = 29) were prepared to cover their own costs.

Patients more often favoured weekly meetings (44%, *N* = 50) than the other options presented: more than once per week (25%, *N* = 29), fortnightly (12%, *N* = 14), monthly or less (15%, *N* = 17) or no preference (4%, *N* = 5). Similarly, 39% (*N* = 45) of patients preferred hour-long meetings rather than alternative options: less than half an hour (3%, *N* = 3), half an hour (16%, *N* = 19), 2 hours (18%, *N* = 21), more than 2 hours (19%, *N* = 22) and no preference (5%, *N* = 6).

With respect to duration of the relationship, the majority felt that it should be open-ended (53%, *N* = 62). Others selected 6 months as a preferred duration (16%, *N* = 18) with less than 10% selecting each of the remaining options available: no preference (*N* = 11), 1 month (*N* = 3), 3 months (*N* = 4), 9 months (*N* = 3), 12 months (*N* = 10) and more than 12 months (*N* = 5).

## Discussion

### Main findings

Reported preferences for befriending schemes showed that a substantial number of patients with SMI would like to take part in a befriending scheme if one were advertised. Patients with lower SQOL were more likely to express a willingness to become a befriendee.

Of those patients willing to take part, approximately 40% preferred weekly, 1-hour long meetings, and 53% would like the relationship to be open ended rather than having a specified duration. However, consensus on the exact type of the preferred befriending was limited, and for most characteristics of both the befriender and the type of relationship there was a considerable variation in responses.

### Strengths and limitations

The present study is, to the authors’ knowledge, the first to explicitly focus on patient preferences for befriending, in keeping with the growing focus on patient perspectives [10, 11]. The sample size offered sufficient statistical power to detect major differences between groups either willing or unwilling to take part in befriending. Further, patients were assessed by experienced, trained researchers through face-to-face meetings, thus contributing to the reliability of the data.

The main limitation of the study relates to the selective nature of the recruitment procedure. The sample was exclusively recruited in East London, and the interviewed patients represent only 32% of those screened and eligible. It is therefore unclear to what extent the findings are generalizable to other populations with SMI.

Similarly, the diagnostic codes used to assess eligibility may have resulted in some participants being included without a SMI. Mild depression, for instance, falls within the diagnostic groups included. However, the low likelihood of such diagnoses being found in CMHT settings and the high proportion of individuals with a psychotic or related disorder lends itself to justifying the overall sample being considered severe. The decision to include a variable sample also allows for greater applicability of the findings to psychiatric populations, which is further supported by the lack of statistical relationship between diagnostic group and willingness to participate in befriending.

The eligibility criteria may have impacted the findings due to exclusion of individuals involved in an ongoing befriending trial. While the overall impact is presumably negligible due to the small number excluded, it is possible that the proportion of people with SMI willing to participate in befriending would have been increased if these individuals had been included. However, the criterion was in place to maintain separation of the samples such that the research findings of the present study and the present trial could contribute to the existing literature independently and inform one another.

A further limitation relates to the potential recall bias for some of the measures used. The Social Contacts Assessment and time use survey necessitate memory of the week prior to the assessment. However, this may have been aided by the presence of a skilled researcher rather than self-administration of the questionnaire.

### Comparison with literature

The present findings for patients’ SQOL [19], loneliness and number of social contacts [15] are consistent with previous studies of similar diagnostic groups, thus suggesting that the results may be applicable beyond the present sample. However, existing literature on befriending schemes is limited to volunteer experiences and organisational features of befriending, as these are typically the limiting factor to expanding such services [20]. As such, little is available for comparison with the present findings with regard to patient preference.

Systematic reviews of existing befriending schemes offer insight into the character of befriending relationships from the perspective of the organisation running the scheme [2, 9]. Through their systematic search, Siette and colleagues [9] found 14 befriending trials. All involved one-to-one interactions between the volunteer befriender and patients. The median frequency and duration of each session was approximately weekly, for 1 hour, and lasted around 3 months (the maximum allowed was 12 months). These figures would generally indicate agreement with preferences expressed by the current sample. Similarly, Hallet and colleagues [21] noted that many schemes they reviewed required a minimum time commitment from volunteers (typically 12 months). However, the real-life duration exhibited great variation, reflecting patient preferences for open-ended relationships. This variation appears to reflect deviation from the prescribed model by the befriender pair, rather than flexibility of the schemes. The frequency of contact, too, varied largely between organisations and befriender pairs within those organisations, with some meeting for 5 hours per week and others only 4 hours per month. While the apparent variability between schemes may cater to the varied patient preferences reported here, it is unclear whether patients have sufficient access to their preferred befriending scheme, or if existing schemes offer the flexibility to adapt to individual preferences.

Consideration should also be given to the role of befriending in relation to peer support. Two-thirds of the current sample would like a befriender with experience as a patient in mental health care. While this points to an overlap between the services in terms of patient preferences, the similarities may not extend to volunteer characteristics and motivations, or the overall aim of the services. However, a recent survey of volunteer befrienders has provided data on volunteer befriender characteristics (Toner, S., Cassidy, M., Chevalier, A., Farreny, A., Leverton, M., Pinto da Costa, M., & Priebe, S. (2018). Preferences for befriending schemes: a survey of patients with severe mental illness. BMC psychiatry, 18(1), 64. 10.1186/s12888-018-1643-9). Around one-third of the volunteer sample had experience of psychiatric treatment, thereby suggesting that volunteer befriender characteristics may be consistent with patient preferences. Further, the preferences expressed by patients may be applicable to and provide insight for other community-based schemes akin to befriending.

### Implications

Whilst socio-demographic characteristics of patients were not significantly linked with the willingness to participate in befriending schemes, lower SQOL may be a relevant factor when considering patients’ likelihood to take part in befriending schemes. As such, it appears that patients with greater inclination to seek out a befriender may also be those who are most in need for such interventions, and perhaps garner the greatest benefit as a result (e.g. improvement in overall SQOL). Similarly, almost half of those expressing unwillingness to participate did so because they felt the scheme was not relevant to their social needs. While it is possible that this lack of desired social interaction is reflective of the negative symptoms of schizophrenia, the results suggest that subjective quality of life remains higher in this unwilling group despite the potential for negative symptoms influencing responses. Although it appears that this group remains difficult to reach through social interventions such as befriending, they represent only a small proportion of the present sample with SMI. Thus, befriending schemes are likely to be taken up by those patients for whom they have been designed, which may be seen as reassuring and encouraging. Future research should establish to what extent befriending schemes can be effective in improving the SQOL of people with SMI.

While patient preferences are variable, they highlight an important consideration for befriending schemes: rigid prescriptive structures for the relationship between a volunteer and patient may be inappropriate or unhelpful. As such, schemes should aim to develop a sufficiently flexible structure to accommodate individual preferences. A useful default structure, potentially for those unsure about these schemes or requiring some initial guidance would be the more commonly selected weekly, 1-hour sessions outside their home.

Finally, the low proportion of patients with prior knowledge of befriending should be considered in the interpretation of the findings. Although patients were given a brief description of befriending before completing the survey, it is unclear whether a more comprehensive understanding and wider familiarity with such schemes would impact upon the expressed willingness to take part.

## Conclusion

The findings suggest that a significant proportion of patients with SMI are interested in befriending. In particular, those with lower SQOL were more likely to want a befriender. The findings therefore highlight that patients with the greatest need for befriending may also be those most likely to take it up and potentially benefit from it. Further, the variability in patient preferences for befriending suggests that if befriending schemes are to successfully relieve social isolation in mental health populations, a flexible design may be more appropriate than a one-size-fits-all approach.

## Abbreviations

MANSA: Manchester short assessment of quality of life
SMI: Severe mental illness
SQOL: Subjective quality of life

## Authors’ information

ST, MC, AC and ML are researchers with a psychology background. AF is a post-doctoral researcher. MPC is a clinical psychiatrist and PhD candidate. SP is clinical-academic psychiatrist.
